# Correspondence

**Published:** 1878-08

**Authors:** 


### Correspondence.

---

BUFFALO, July 29, 1878.

*Editors Buffalo Medical Journal:*

I send you for publication the inclosed letters. The matter, perhaps, has already received all the attention that it demands. The letters are of interest, however, as showing the composition of the "Boudior" Medicines, and the method taken by their proprietor to advertise them.

Very truly yours,

JAMES P. WHITE.

---

NEW ORLEANS, July 15, 1878.

DEAR SIR:—Some time ago you wrote me about an impostor of your State who had made victims of us both. I wrote you at the time that I had an indistinct recollection (I think), or that I not unfrequently received letters of the kind alluded to. Looking over my old papers for other purposes, have found this man Cronyn's letter, and I enclose it to you to make such use of it as you see fit. My recollection now is that I did write a polite line to the fellow, saying that his prescriptions were probably good. I did this because I supposed he was a gentleman, and I wished to treat him politely—not because I gave the matter any serious thought.

Very truly,

D. WARREN BICKNELL.

PROF. WHITE, M. D., Buffalo.

---

DUNKIRK, Chautauqua Co., N. Y.,
14th May, 1877.

DEAR DOCTOR:—In my female practice I have been prescribing the enclosed combination of medicines as an injection, in Leucorrhœa and Ulceration (simple) of the Uterus, varied in strength according to the severity of the case.

Acidi Carbolici,
Liq. Plumbi Subacet,
Fluid Ext. Papaveris,
Glycerine,
Aqua Rosa.

I have been much interested in your articles in the *Amer. Jour. of Med. Sciences*, and I write to ask your opinion of the above, and also the following tonic medicines, by the stomach, for the relief of the dozen and one complaints always attendant upon such cases:

| | |
|---|---|
| Tinct. Ferri Mur. | ʒiii |
| Tinct. Nucis Vomica, | ʒii |
| Tinct. Calumbo, | ℥ii |
| Aqua Cinnamoni, | ℥ii |
| Syrupi Simplicis, | q. s. |
| Ft. Mist., | ℥viii |

Signa.—One tablespoonful three times a day.

Very respectfully,

WILLIAM CRONYN.

D. WARREN BICKNELL, M. D.,
Prof. of Obstetrics and Diseases of Women,
Charity Hospital Medical College,
New Orleans.

———:o:———

The Journal of Inebriety mentions two cases in which dipsomania was caused by tape-worm. So soon as the animal was dislodged all craving for alcohol disappeared.

———:o:———

The Boston Medical Library has recently received the library of the late Dr. Edward Warren, of Boston, consisting of two hundred bound volumes and six hundred valuable pamphlets.

